# Acute hyperglycemia impairs IL‐6 expression in humans

**DOI:** 10.1002/iid3.97

**Published:** 2016-01-19

**Authors:** Matthew P. Spindler, Alvin M. Ho, David Tridgell, Marli McCulloch‐Olson, Vivian Gersuk, Chester Ni, Carla Greenbaum, Srinath Sanda

**Affiliations:** ^1^ The Diabetes CenterUCSF School of MedicineSan FrancicscoCalifornia94143USA; ^2^ The Diabetes Research ProgramBenaroya Research InstituteSeattleWashington98040USA

**Keywords:** Hyperglycemia, IL‐6, IL‐17, metabolism, monocyte

## Abstract

Normal glucose metabolism is critical to immune function but the effects of short‐term hyperglycemia on immunity are not well described. To study this phenomenon, we induced hyperglycemia in healthy subjects for 2 h with intravenous dextrose and octreotide. An RNA‐seq analysis of whole blood RNA demonstrated alterations in multiple immune pathways and transcripts during acute hyperglycemia including decreased transcription of IL‐6, an important component of both innate and adaptive immune responses. Additional in vitro studies of human peripheral blood mononuclear cells (PBMCs) exposed to high glucose confirmed decreased IL‐6 expression, most prominently in CD14^+^CD16^+^ intermediate monocytes. Hyperglycemia also reduced IL‐17A expression suggesting further impairment of immune responses during acute hyperglycemia. These findings demonstrate multiple defective immune responses in acute hyperglycemia and suggest a novel role for intermediate monocytes as metabolically sensitive innate immune cells.

## Introduction

There is increasing evidence that the immune system can influence metabolism 1, 2, 3, 4. However, the effects of alterations in host metabolism on immune function may also be clinically relevant. Acute hyperglycemia is commonly encountered in critically ill patients and is associated with increased mortality 5, 6, 7, 8, 9. Critically ill subjects, particularly those with sepsis or multi‐organ failure, can also present with dysfunctional immune responses termed “immune paralysis;” however, it is unclear if there is a connection between hyperglycemia and impaired immune responses 10, 11. In addition, brief episodes of hyperglycemia are noted on oral glucose tolerance tests in subjects at risk for developing type 1 diabetes prior to the onset of clinical symptoms. These episodes of acute hyperglycemia increase rates of progression to type 1 diabetes but the mechanism for this additive risk remains unclear 12.

Previous studies have described immune responses during acute hyperglycemia and have focused on expression of IL‐6. Using THP‐1 cell lines, investigators have shown that in vitro hyperglycemia increased IL‐6 expression 13. In humans, data suggest that plasma IL‐6 levels are increased in critically ill subjects admitted with hyperglycemia but these levels were measured randomly and not during controlled, provocative metabolic testing 14. However, when oral glucose tolerance testing was performed in humans with impaired glucose tolerance, there was a trend for decreasing plasma IL‐6 protein levels during mild hyperglycemia 15. Given the clinical importance of acute hyperglycemia and the conflicting data regarding its effects on immune function, we chose to address this problem by combining in vivo transcriptome analysis and immune phenotyping of primary human cells.

## Methods

A full description of the methods is provided in the supplementary section.

### Subjects and study approval

For the glucose‐octreotide protocol, 10 healthy control subjects were recruited from the control registry of the Translational Research Program at the Benaroya Research Institute at Virginia Mason Medical Center. All subjects were negative for islet autoantibodies and had no personal or family history of diabetes. The registry, as well the glucose‐octreotide protocol, had local institutional review board approval prior to enrollment. Sixty percent of the subjects were female; the average age was 28.3 years ± 5.9. All subjects provided written informed consent prior to study procedures.

For all in vitro studies, samples from the control subject repository at the Benaroya Research Institute were used. Subjects had donated blood samples for research purposes. All were negative for diabetes autoantibodies. All subjects had provided informed consent prior to blood donation. Samples were used once and were not used in multiple in vitro experiments.

### Statistics

For the RNA‐seq analysis, we calculated the differential expression between pair‐wise FPKM values at *T* = 0 and *T* = 120. Linear models were fitted for each gene using the Bioconductor “limma” package in R 16, 17. Moderated t‐statistics, fold‐change, and the associated p‐values were calculated for each gene. The FDR (false discovery rate) was computed for each gene using the Benjamini–Hochberg method 18. Over‐represented pathways were identified using the program GO Elite 19. Only pathways with a *P*‐value < 0.01 after correcting for multiple comparisons were determined to be significantly over‐represented. All other analyses of ELISA, electrochemiluminescence, and flow cytometry data were done with Prism (GraphPad software) using ANOVA comparisons for multiple groups and nonparametric pair‐wise testing to determine statistical significance.

## Results and Discussion

**Figure 1:**
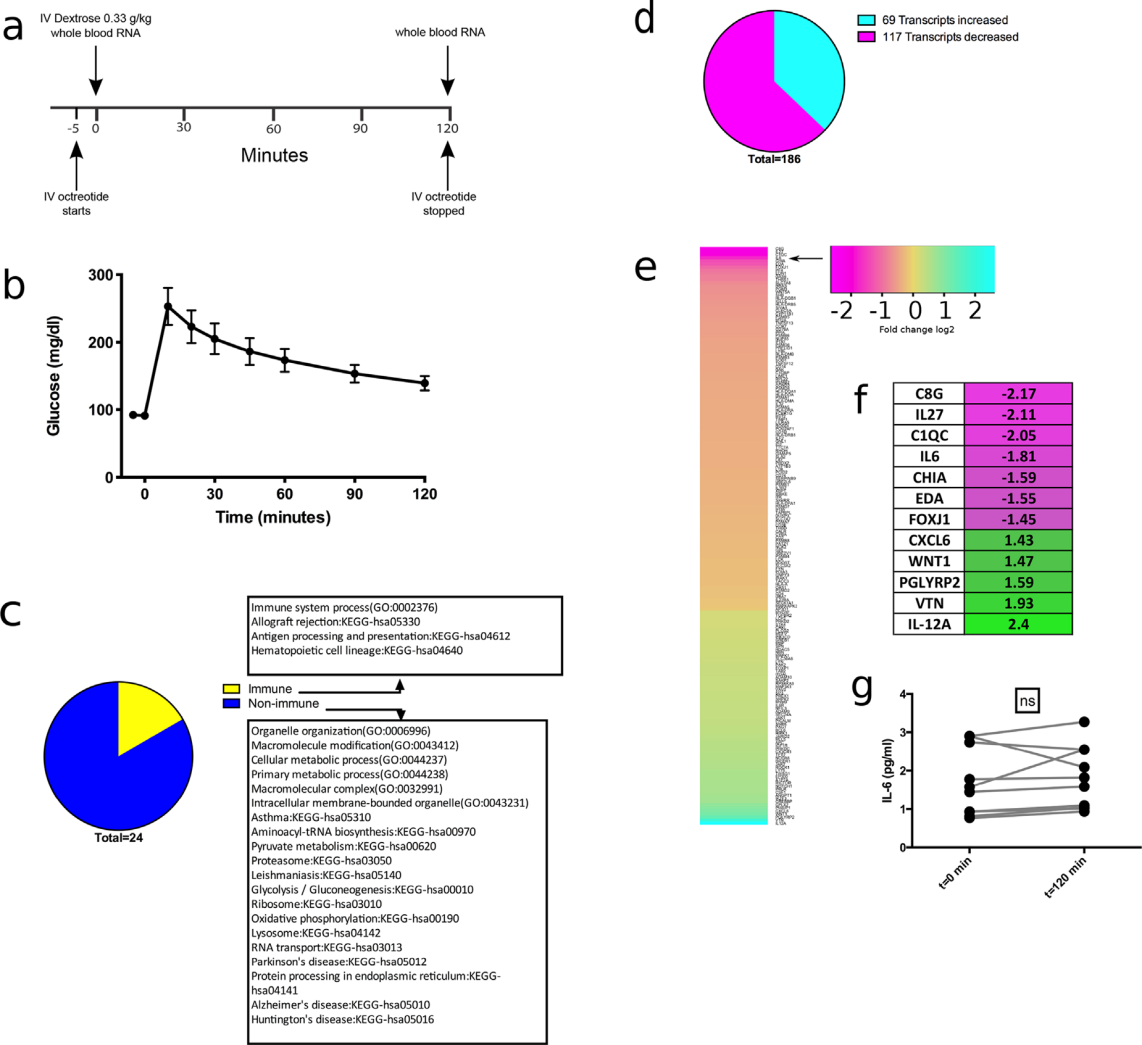
Suppression of immune gene expression during in vivo acute hyperglycemia in human subjects. (a) Clinical protocol for 10 healthy control subjects. Participants were admitted to the clinical research center and received a bolus and continuous infusion of octreotide for 120 min. Dextrose bolus occurred at *t* = 0 min and collection of whole blood RNA occurred at *t* = 0 and *t* = 120 min. (b) Glucose excursion over 2 h in all 10 subjects with standard deviations. (c) Pathways significantly altered (*P*‐value < 0.01 via Benjamin–Hochberg method) between the whole blood RNA‐seq profiles at *t* = 0 and *t* = 120 min. (d) Proportion of transcripts that increased and decreased in the immune system process pathway (GO: 0002376). (E) Heatmap showing fold changes (in log 2) in the immune system process pathway (GO: 0002376). Arrow indicates *IL‐6* expression in RNA‐seq analysis. (f) Transcripts from (e) with the linear fold change > 1. (g) Serum IL‐6 levels did not change during the 2‐h protocol.

We began studying the effects of acute hyperglycemia on immune function with an in vivo clinical experiment. Ten healthy subjects without diabetes received intravenous octreotide (to suppress endogenous insulin secretion and maximize short‐term hyperglycemia) followed by a single bolus of intravenous dextrose (Fig. 1A). Glucose levels peaked at 10 min post‐dextrose bolus (mean peak glucose 253 mg/dL ± 27.3) and then declined (Fig. 1B). Whole blood RNA was collected prior to intravenous dextrose and at 120 min post‐dextrose bolus. All subjects tolerated the octreotide and glucose bolus well without significant adverse events. A separate protocol only using octreotide was not performed as octreotide‐only infusions have not been found to alter cytokine levels 20.

An RNA‐seq analysis was performed on the whole blood RNA comparing the baseline and 120‐min RNA samples. A pathway enrichment analysis demonstrated 24 statistically significantly altered pathways during acute hyperglycemia of which 4 were immune based pathways (Fig. 1C). We examined the immune system process pathway (GO: 0002376, *P*‐value = 0.002 after correcting for multiple hypothesis testing) as it contained the largest number of differentially expressed immune transcripts in our analysis. We observed that the majority of transcripts, including *IL‐6*, decreased with acute hyperglycemia (Fig. 1D and 1E). We focused on validating the *IL‐6* signature given the magnitude of its observed fold change in relation to other transcripts, its importance in innate and adaptive immunity, and its association with hyperglycemia in previous studies (Fig. 1F). We analyzed serum from patients in the hyperglycemia protocol, collected at the same time as the RNA, and observed no changes in IL‐6 protein levels with the acute hyperglycemia (Fig. 1G). Given the short duration of the clinical intervention, we decided to model acute hyperglycemia in vitro to allow us to observe changes in IL‐6 protein levels with longer periods of hyperglycemia.

**Figure 2:**
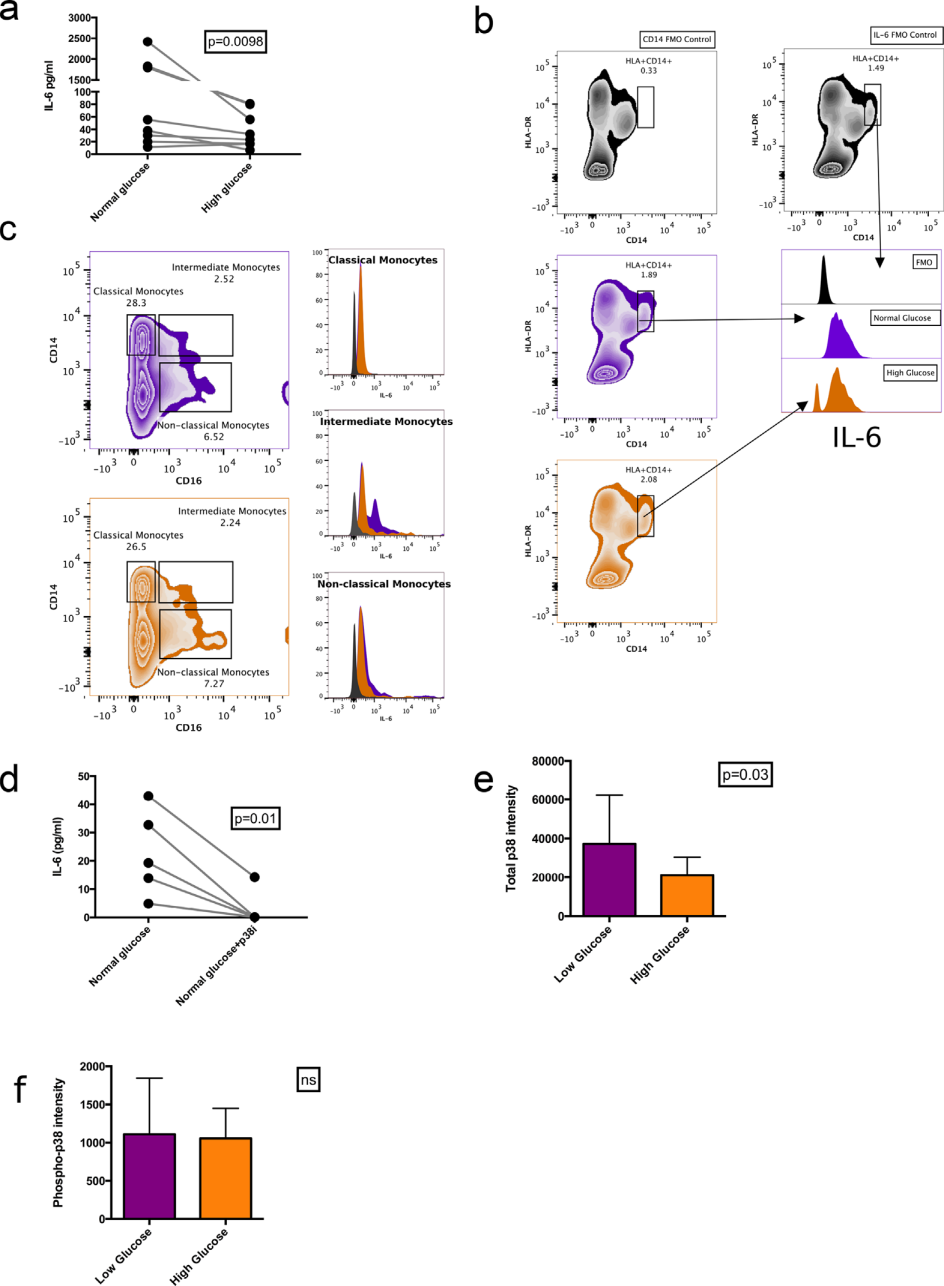
Acute hyperglycemia decreases IL‐6 expression in intermediate monocytes. (a) IL‐6 levels in PBMC culture supernatants after 5 h from 10 individuals analyzed with a Wilcoxon matched‐pairs sign rank test (b) Representative flow plots showing decreased intracellular IL‐6 in HLA‐DR^+^CD14^+^ monocytes with acute hyperglycemia. Whole PBMCs were gated for HLA‐DR^+^CD14^+^. Double positive cells were selected (circles) based on fluorescence minus one (FMO) control for both HLA‐DR and CD14. The FMO control for CD14 expression is shown in gray. Normal glucose = purple, high glucose = orange, fluorescence minus one (FMO) IL‐6 control = grey. (c) Representative flow plots showing monocyte subsets based on CD14 and CD16 staining. Gates drawn based on FMO controls (not shown). Normal glucose = purple and high glucose = orange. Histograms of intracellular IL‐6 staining in monocyte subsets from sample shown in C. Significant IL‐6 is only seen in intermediate monocytes and decreases with acute hyperglycemia. (d) Whole PBMC culture supernatants of five individuals exposed to normal glucose media and normal glucose media with a p38 inhibitor, SB203580, showed reduced IL‐6 expression (testing with a Wilcoxon rank sign test). (e) Whole PBMCs (*n* = 5) exposed to normal and high glucose show reduced mean p38 protein expression (testing with a Wilcoxon rank sign test, standard deviation bars shown). (f) Whole PBMC (*n* = 5) exposed to high glucose showed no significant change in mean phospho‐p38 levels (testing with a Wilcoxon rank sign test, standard deviation bars shown).

We next studied another cohort of 10 healthy control individuals (see methods) not studied in our hyperglycemia protocol. Frozen human peripheral mononuclear cells (PBMC) samples were equally divided into a normal glucose (100 mg/dL) environment and a high glucose (450 mg/dL) environment for 5 h. Cell numbers were equivalent in both cultures. Cell culture supernatants were harvested and analyzed for IL‐6 by ELISA. IL‐6 levels significantly decreased in high‐glucose environments (Fig. 2A). Of the 10 subject samples analyzed under these conditions, 9 showed reduced IL‐6 expression with hyperglycemia. Exposing PBMCs to osmotically equivalent amounts of mannitol did not affect IL‐6 expression (Supplementary Fig. S1).

We next analyzed the cell types secreting IL‐6 by flow cytometry after the 5‐h culture described above. When exposed to high glucose, monocytes (CD14^+^HLA‐DR^+^) demonstrated decreased IL‐6 expression, correlating with the ELISA data (Fig. 2B). Purified T‐cells did not show significant IL‐6 expression by ELISA or flow cytometry in our 5‐h culture experiment (Supplementary Fig. S2).

As only a subset of CD14^+^ cells seemed to respond to high glucose, we divided the monocyte population into subsets based on CD14 and CD16 staining to identify classical (CD14^+^CD16^−^), non‐classical (CD14^−^CD16^++^), and intermediate monocytes (CD14^+^CD16^+^) 21, 22. Whole PBMCs were gated for CD14 and CD16 with fluorescence minus one (FMO) controls. We observed that only intermediate monocytes (CD14^+^CD16^+^) showed significant intracellular IL‐6 that decreased when cells were cultured in high glucose compared to normal glucose (Fig. 2C).

To investigate the mechanism of hyperglycemic‐induced IL‐6 suppression, we studied the p38 mitogen‐activated protein kinase (p38MAPK) given its importance in regulating IL‐6 expression 23, 24, 25. A previous study had shown that hyperglycemic environments impair p38MAPK activity in keratinocytes 26. A modest reduction in the mRNA of the p38 gene, *MAPK11*, was seen in the RNA‐seq data from the in vivo clinical study (data not shown). No other isoform of p38 was detectable in the RNA‐seq analysis. Blockade of p38 activity with the inhibitor SB203580 decreased in vitro IL‐6 secretion from whole PBMCs after 5 h confirming the importance of p38 in IL‐6 expression (Fig. 2D). SB203580 only inhibits the kinase activity of p38MAPK but does not prevent its phosphorylation 27. Using an electrochemiluminescence assay to quantify total and phosphorylated p38 protein, we observed that hyperglycemia did not alter the phosphorylation status of p38MAPK but did reduce total amounts of p38 (Fig. 2E and 2F). These data suggested that hyperglycemia reduces p38 expression, but not phosphorylation, leading to reduced IL‐6 expression.

**Figure 3:**
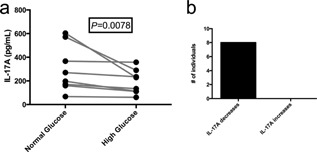
Acute hyperglycemia decreases IL‐17A expression. (a) IL‐17A measured by ELISA from cell culture supernatants in PBMC samples (*n* = 8) with in vitro acute hyperglycemia. (b) Number of samples that decreased IL‐17A expression with hyperglycemia.

Finally, we sought to determine if hyperglycemia‐mediated IL‐6 suppression could alter downstream adaptive immunity given the importance of IL‐6 in differentiating naïve T‐cells into TH17 cells 28. We hypothesized that a high‐glucose environment would reduce IL‐17A expression in an IL‐6‐dependent fashion. PBMC samples from another eight control individuals were equally divided into normal and high‐glucose wells for 5 h followed by non‐specific stimulation with PMA/ionomycin for an additional 12 h. Exposure of primary PBMCs to a high‐glucose environment reduced IL‐17A expression by ELISA compared to a normal glucose environment (Fig. 3A and 3B).

## Discussion

Together, these data characterize the immune responses to short‐term acute hyperglycemia in humans. We show that short‐term hyperglycemia decreases IL‐6 expression in CD14^++^CD16^+^ intermediate monocytes and IL‐17A expression. These findings may have clinical relevance to the “immune paralysis” associated with critical illness and the development of type 1 diabetes.

These findings also add to our growing knowledge that intermediate monocytes have a distinct metabolism from other monocyte subsets and may serve as circulating immuno‐glucose sensors 29. Future studies will evaluate inherent metabolic differences between human monocyte subsets and mechanisms of hyperglycemic suppression of p38 expression. Also, while our in vitro data explored the inhibition of IL‐6 during of acute hyperglycemia, our in vivo RNA‐seq data from the glucose‐octreotide protocol suggest that multiple other immune transcripts increase with hyperglycemia including IL‐12A, which is important in TH1 development. It is probable that the effects of hyperglycemia on adaptive immune cells are complex and will require additional studies to fully elucidate them.

Our observation of reduced IL‐6 expression with hyperglycemia differs from previous publications. Methodological differences such as samples types and culture conditions used most likely account for these differences. We believe, however, that our in vivo RNA‐seq data combined with our in vitro data from primary human cells are valid models of immune responses during acute hyperglycemia. It is not clear if these data are readily applicable to chronic forms of hyperglycemia such as types 1 and 2 diabetes but an ongoing clinical trial with a monoclonal antibody against IL‐6 in type 1 diabetes (NCT02293837) may be informative.

Despite these limitations, we believe these data provide valuable mechanistic data relevant to immune function. It adds to our knowledge of human monocyte subsets and improves our understanding of the interaction between glucose metabolism and immunity.

## Conflict of Interest

The authors report no conflicts of interest relevant to this publication.

## Supporting information

Additional supporting information may be found in the online version of this article at the publisher's web‐site.

**Figure S1**. Increased osmolarity with mannitol does not affect IL‐6 secretion from whole PBMCs after 5‐h culture (*n* = 3).
**Figure S2**. Monocytes and T‐cells from two individuals were isolated from whole PBMC samples using magnetic bead separation.
