# Supplementary material for: Acute hyperglycemia impairs IL‐6 expression in humans
Source: Immun Inflamm Dis. 2016 Jan 19;4(1):91–7. doi: 10.1002/iid3.97 (PMC4768063; doi:10.1002/iid3.97)
Supplement: Supplementary file 1 — Figure S1. Increased osmolarity with mannitol does not affect IL‐6 secretion from whole PBMCs after 5‐h culture (n = 3). Figure S2. Monocytes and T‐cells from two individuals were isolated from whole PBMC samples using magnetic bead separation. [file IID3-4-91-s001.docx]

**Supplementary Methods**

*Glucose-octreotide protocol*

Subjects arrived fasting to the Benaroya Research Institute CRC at Virginia Mason Medical Center.  Two IVs were placed and patients received a bolus of 25 ug of octreotide.  Then a continuous infusion of octreotide at 0.48 ug/min was started.  After 5 minutes, an IV bolus of dextrose (0.33 g/kg) was administered.  Blood samples for glucose, insulin, C-peptide, and mechanistic blood samples including whole blood RNA were taken at time points: -5 (pre-octreotide bolus), 0 (immediately prior to dextrose bolus) and 10, 20, 30, 45, 60. 90, and 120 minutes post dextrose.  Vital signs were monitored every 30 minutes post dextrose bolus.  After 120 minutes, the octreotide infusion was discontinued.    The IVs were removed; the subject was fed and discharged from the CRC in stable condition.

*Whole blood RNA extraction*

Blood was collected in PAXgene Blood RNA tubes and frozen.  Total RNA was extracted using the MagMAX for Stabilized Blood Tubes RNA Isolation Kit, compatible with PAXgene Blood RNA Tubes (Life Technologies), and depleted for globin mRNA using the GLOBINclear human kit (Life Technologies).  RNA-seq libraries were constructed from globin-reduced RNA using the TruSeq RNA Sample Prep Kit (Illumina) with selection of poly-adenylated transcripts.  Libraries were clustered on a flowcell using the TruSeq Paired-end Cluster Kit, v3 on a cBot clustering instrument (Illumina), followed by paired-end sequencing on a HiScanSQ (Illumina) for 50 cycles in either direction.

After the run was completed, image files were processed by Illumina Off-Line Basecaller (OLB) and subsequently by CASAVA for base calling and demultiplexing.  The resulting FASTQ files were then aligned to UCSC human genome release hg19 using Bowtie and TopHat ([29](#_ENREF_29), [30](#_ENREF_30)). We determined quantitation of gene expressions (FPKM, Fragments Per Kilobase Of Exon Per Million Fragments Mapped) by Cufflinks from aligned paired-end reads ([31](#_ENREF_31), [32](#_ENREF_32)).

*Cell culture and cytokine analysis*

Frozen PBMCs were thawed and washed in hybridoma media (Gibco 12045-084) supplemented with 5% human AB serum, nonessential amino acids, and pyruvate. Split cultures of cells (~5x10^6^ cells/well) were cultured in normal glucose (100 mg/dl) DMEM media (Cellgro 10-014-CV) or high glucose (450 mg/dl) DMEM media (Cellgro 10-013-CV) with 5% human AB serum, nonessential amino acids, and pyruvate. Mannitol (Sigma-Aldrich 63559) was used to bring tonicity of low glucose DMEM media to a similar level as high glucose media. PMA/ionomycin (eBioscience 00-4975-03) was used per manufacturer’s instructions. Serum cytokines were analyzed using an ultrasensitive serum electrochemiluminescence assay from MesoScale Discoveries. Cell culture supernatants were isolated, frozen, and cytokines analyzed by ELISA using antibodies for IL-6 and IL-17A (BioLegend).

*p38 quantification*

Frozen PBMCs were thawed and cultured for 5 hours as described above with and without a p38 inhibitor, SB203580 (Cell Signaling #5633). Cells were lysed using proprietary reagents from Mesoscale Discovery and analyzed using a multi-spot electrochemiluminescence assay (K15112D-1) and analyzed on a QuickPlex SQ 120 machine. *MAPK11* primers were as follows: (forward) ACCTCACTGCTCAATCTCC and (reverse) ACCTCACTGCTCAATCTCC.

*Flow cytometry*

Cells obtained from cell culture were washed in buffer containing PBS, 1%BSA and 0.1% sodium azide. Cells were incubate with surface fluorophore antibodies for 30 minutes at room temperature and subsequently washed in buffer containing PBS, 1%BSA and 0.1% sodium azide. Surface staining was done using antibodies from eBiosciences (CD14) and BioLegend (CD16, CD4, CD45RO, and HLA-DR). Intracellular staining was done using the FoxP3 Permeabilization Buffer kit from BioLegend (421403)**.**  Intracellular antibodies from eBiosciences (IL-6) and BioLegend (IL-17A) were used.

All samples were run on the same flow cytometer (LSR II) using automated compensation. Single stain controls with compensation beads (eBioscience 01-2222-42) were used. Gates were drawn based on fluorescence minus one controls (FMO) using cells exposed to similar conditions ([33](#_ENREF_33)). All data was analyzed using the FlowJo software package.

**Supplementary Figures.** Figure 1. Increased osmolarity with mannitol does not affect IL-6 secretion from whole PBMCs after 5-hour culture (n=3). Figure 2. Monocytes and T-cells from two individuals were isolated from whole PBMC samples using magnetic bead separation. Cell populations were cultured for 5 hours in low and high glucose. T-cells do not exhibit detectable IL-6 secretion compared to monocytes.
